# Impact of underlying liver disease on unresectable hepatocellular carcinoma treated with immune checkpoint inhibitors

**DOI:** 10.1038/s44276-024-00038-w

**Published:** 2024-01-29

**Authors:** Y. Linda Wu, Sarah Cappuyns, Amanda Loh, Sean Sun, Sara Lewis, Max W. Sung, Myron Schwartz, Josep M. Llovet, Deirdre J. Cohen

**Affiliations:** 1 Division of Hematology and Medical Oncology, Tisch Cancer Institute, Icahn School of Medicine at Mount Sinai, New York, NY USA; 2 Division of Hematology and Oncology, Herbert Irving Comprehensive Cancer Center, Columbia University Irving Medical Center, New York, NY USA; 3 Mount Sinai Liver Cancer Program, Division of Liver Diseases, Tisch Cancer Institute, Icahn School of Medicine at Mount Sinai, New York, NY USA; 4 Digestive Oncology, Department of Gastroenterology, UZ Leuven/KU, Leuven, Belgium; 5 Department of Diagnostic, Molecular and Interventional Radiology, Icahn School of Medicine at Mount Sinai West, New York, NY USA; 6 Department of Diagnostic, Molecular and Interventional Radiology, Icahn School of Medicine at Mount Sinai, New York, NY USA; 7 Translational Research in Hepatic Oncology, Liver Unit, IDIBAPS, Hospital Clinic, University of Barcelona, Barcelona, Spain; 8 Institució Catalana de Recerca i Estudis Avançats (ICREA), Barcelona, Spain

## Abstract

**Background:**

Immune checkpoint inhibitors (ICIs) are standard therapy for unresectable HCC, but many patients do not respond. Non-viral HCC, particularly non-alcoholic steatohepatitis (NASH), have been implicated in ICI resistance.

**Methods:**

We reviewed 288 patients with unresectable HCC who received ICI from 1/2017 to 12/2021. The overall survival (OS), progression-free survival (PFS), and objective response rate (ORR) between patients with viral HCC and non-viral HCC were compared using the full and Child Pugh (CP) class A only cohorts.

**Results:**

In total, 206 patients (71.5%) had viral HCC (most HCV), and 82 patients had non-viral HCC. Non-viral HCC was associated with worse OS (HR 1.6, 95% CI: 1.1–2.1, *P* = 0.006) and PFS (HR 1.5, 95% CI: 1.2–2, *P* = 0.002) in univariate but not multivariate analyses. For the CP class A cohort, non-viral HCC was independently associated with worse OS (HR 1.8, 95% CI: 1.2–2.7, *P* = 0.005) and PFS (HR 1.9, 95% CI: 1.3–2.7, *P* < 0.001). Viral HCC and CP class A liver disease was associated with better ORR than non-viral HCC (38% vs. 16%, *P* = 0.001).

**Conclusions:**

Following ICI treatment, non-viral HCC correlated with worse OS, PFS, and ORR than viral HCC, particularly in patients with preserved liver function.

## Introduction

Hepatocellular carcinoma (HCC) is a leading cause of cancer-related mortality and rising in incidence worldwide [1]. Systemic therapy is the mainstay of treatment for patients with advanced or unresectable HCC, and until recently, it consisted of multikinase inhibitors like sorafenib, with limited efficacy and high toxicity [2, 3]. Now, both the combinations of atezolizumab, a programmed death-ligand 1 (PD-L1) inhibitor, plus bevacizumab, a vascular endothelial growth factor (VEGF) inhibitor [4, 5], and durvalumab, another anti-PD-L1 antibody, plus tremelimumab, a cytotoxic T lymphocyte-associated antigen 4 (CTLA-4) inhibitor, have demonstrated improved efficacy over sorafenib as first-line treatments for unresectable HCC [6]. Accordingly, immune checkpoint inhibitors (ICIs) have become standard front-line therapy. Nevertheless, approximately 20-40% of patients experience primary resistance to these regimens [4–6], underscoring the need to identify patients most likely to benefit from ICIs.

Both hepatitis B virus (HBV) and hepatitis C virus (HCV) infections, despite viral clearance with antiviral medications, are major risk factors for the development of cirrhosis and HCC [7, 8]. Increasingly, non-alcoholic steatohepatitis (NASH) has emerged as an important etiology for HCC, especially in the West [8, 9]. Recently, preclinical models showed that NASH-induced HCC may have reduced response to ICIs via aberrant T cell activation and impaired immune surveillance, and analyses of phase III clinical trials suggest that NASH-driven HCC lead to worse outcomes with ICI treatment than viral-induced HCC [10]. However, other recent meta-analyses found no link between etiology and objective response rate (ORR) and yielded inconclusive results regarding overall survival (OS) [9, 11, 12]. Evidently, more studies are needed to elucidate the effect of viral infection on outcomes with ICI treatment.

Response to immunotherapy depends on a complex interplay of immunosuppressive and immune-promoting signals in the tumor microenvironment. Viral antigens expressed by tumor cells have been shown to act as potent antigens to enhance response to ICI [12]. Underlying viral infections, such as human papilloma virus-driven squamous cell carcinoma of the head and neck and Epstein-Barr virus-related gastric cancer, have been correlated with improved ICI response [13–15]. Therefore, the tumor immune microenvironment of viral- and non-viral induced cancers are likely distinct. In HCC, chronic viral infections stimulate both pro-inflammatory innate and aberrant adaptive immune responses that fail to clear these viruses, promoting tumorigenesis [1]. However, the impact of HCC etiology on ICI treatment remains unclear, revealing an area of unmet need.

Our study aimed to compare the survival outcomes following ICI therapy of patients with viral and non-viral HCC using a real-world, diverse population from a large tertiary care institution, with particular attention to the effect of NASH. Growing evidence suggests that anti-angiogenic agents (AAs) may be synergistic with ICIs, likely through vascular normalization that improves drug delivery and promotion of antitumor immunity [1]. Additionally, locoregional therapy (LRT) may be synergistic with ICIs as it promotes immunogenic tumor cell death, releasing antigens that stimulate pro-inflammatory cytokines and prime antitumor lymphocytes [16]. We thus also evaluated the efficacy of concurrent LRT and AAs in combination with ICIs.

## Methods

### Patient population

The study included patients who received front-line ICI, including nivolumab, pembrolizumab, and atezolizumab, for advanced, unresectable HCC at the Mount Sinai Health System from January 2017 to December 2021. The study population had a radiologic or histologic diagnosis of HCC in accordance with American Association for the Study of Liver Disease guidelines [17]. Patients included in the study also had Child Pugh (CP) class A or B liver function and Eastern Cooperative Oncology Group (ECOG) performance status (PS) 0 to 2. Patients who underwent liver transplantation after immunotherapy were excluded. The decision to treat with ICI with or without LRT was made at the treating physician’s discretion according to routine clinical practice, based on current practice guidelines, institutional standards, and multidisciplinary tumor board consensus. Baseline esophagogastroduodenoscopies were also performed at each clinician’s discretion. In this prospectively maintained database of 414 patients, 288 met inclusion criteria for analysis (Supplemental Fig. 1).

### Study design

Patient demographic and clinical data, including Barcelona Clinic Liver Cancer (BCLC) stage, CP class, ECOG PS, alpha fetoprotein (AFP) level, presence of cirrhosis (radiologically or rarely histologically diagnosed), presence of extrahepatic metastases, presence of portal venous tumor thrombosis (PVTT), etiology of liver disease, receipt of LRT, follow-up, and vital status, were collected retrospectively. Baseline data were defined as the time of ICI initiation. Patient response to therapy was evaluated using computerized tomography and/or magnetic resonance imaging approximately every 3 months during treatment. All responses were determined using modified Response Evaluation Criteria in Solid Tumors (mRECIST) criteria for HCC. While both RECIST v1.1 and mRECIST are recommended for patients with advanced HCC undergoing systemic therapy, mRECIST has the advantage of accounting for effects of LRT as well as clinical events due to natural progression of chronic liver disease [18, 19]. All images were reviewed by dedicated radiologists.

The primary objective was to compare the OS of patients with viral HCC with the OS of patients with non-viral HCC upon ICI treatment, and to specifically explore the impact of NASH, where data is scarce. Viral HCC was defined as HBV or HCV infection, based on laboratory and clinical parameters, including hepatitis B core antibody positivity, hepatitis B surface antigen positivity, detection of HBV DNA by polymerase chain reaction (PCR), HCV antibody positivity, detection of HCV DNA by PCR, and prior or ongoing HBV or HCV treatment. Any patient with HCV or HBV infection was categorized as viral, including patients with mixed etiologies. All others were considered to have non-viral HCC. Patients with NASH were identified based on radiologic report, histopathologic diagnosis, where available, and clinical assessment per routine clinical practice and as previously defined [9].

Secondary objectives included evaluating the effect of viral and non-viral HCC on progression-free survival (PFS), ORR, defined as the proportion of patients with either radiologic complete response (CR) or partial response (PR), and duration of response (DOR), measured from the start of ICI treatment until disease progression if a CR or PR had been achieved. Analyses were conducted for the full cohort and for patients with CP class A liver disease only, and separate analyses were performed to compare patients with viral and NASH-induced HCC. We also assessed the effect of combination ICI and AAs compared to ICI monotherapy. Finally, we investigated the impact of concurrent immunotherapy and LRT, including transarterial chemoembolization (TACE), transarterial radioembolization (TARE), radiofrequency ablation (RFA), and stereotactic body radiation therapy (SBRT).

### Statistical analysis

Patient characteristics were reported descriptively as medians and interquartile ranges (IQRs) for continuous variables and percentages for categorical variables. Variables were compared between viral and non-viral HCC patients using the Wilcoxon rank sum-test for continuous variables and Fisher exact test for categorical variables. OS was defined as the time of ICI initiation until death from any cause, and patients alive at the time of data cut-off were censored at the last follow-up. PFS was calculated as the time from the start of therapy to radiologic or clinical progression or death, whichever occurred first. Patients without progression or death at the time of data cut-off were censored at the last follow-up. Median OS and PFS were estimated using the Kaplan-Meier method and compared between groups using the log-rank test. Uni- and multivariate Cox regression analysis was used to identify parameters associated with OS and PFS between viral and non-viral HCC, as well as viral and NASH-induced HCC. All variables with *P* value < 0.05 in univariate analysis were included in the multivariate analysis, and they are represented using hazard ratio (HR) and 95% confidence intervals (CIs). Statistical analyses were conducted using R statistical software (version 4.0.4), R Project for Statistical Computing using the R packages ‘survival’ (version 3.3.1) and ‘survminer’ (version 0.4.9).

## Results

### Patient characteristics

Baseline characteristics of the 288 patients who met inclusion criteria are reported in Table 1. The median age was 64 years (IQR: 60-70 years). Most patients were male (*N* = 245, 85.1%) and had radiologic or pathologic evidence of cirrhosis (*N* = 250, 86.8%). The most prevalent etiology of HCC was viral (*N* = 206, 71.5%), with the majority due to HCV (*N* = 151, 73.3%) and 27.2% due to HBV. Of the 28.5% of patients with non-viral HCC, 33 (40.2%) were due to alcohol use and 37 (45.1%) were due to NASH, and 7 had mixed non-viral etiologies. At the time of ICI initiation, only 25.2% of those patients with viral HCC had a detectable HBV or HCV viral load. In addition, most patients had CP class A liver disease (*N* = 195, 67.7%), BCLC stage C (*N* = 210, 72.9%), and ECOG PS 0 (*N* = 209, 72.6%). Because of the time points of this study, most patients received front-line nivolumab (*N* = 223, 77.4%), but many received atezolizumab plus bevacizumab (*N* = 47, 16.3%). 20.1% of patients had a history of hepatic resection, and most (59.4%) received prior LRT. A substantial proportion of patients (*N* = 125, 43.8%) received concurrent LRT, defined as LRT within 30 days before or after ICI initiation.

**Table 1:** Patient demographics by etiology (*N* = 288).

			All patients (%)	Viral (%)	Non-viral (%)	*P* value
N			288	206 (71.9)	82 (28.5)	
Age (years), median			64	63.5	68	<0.001*
Male			245 (85.1)	172 (83.5)	73 (89.0)	0.27
Cirrhotic			250 (86.8)	188 (91.3)	62 (75.6)	<0.001*
Diabetic			96 (33.3)	54 (26.2)	42 (51.2)	<0.001*
Etiology:	HBV		56 (19.4)	56 (27.2)	N/A	
	HCV		151 (52.4)	151 (73.3)	N/A	
	EtOH		75 (26.0)	42 (20.4)	33 (40.2)	
	NASH		45 (15.6)	8 (3.9)	37 (45.1)	
	Other		16 (5.6)	14 (6.8)	2 (2.4)	
	None		17 (5.9)	N/A	17 (20.7)	
Measurable Viral Load				52 (25.2)	N/A	
Child Pugh:	A		195 (67.7)	148 (71.8)	47 (57.3)	0.025*
	B		93 (32.3)	58 (28.2)	35 (42.7)	
Extrahepatic Metastasis			105 (36.5)	75 (36.4)	30 (36.6)	1
Portal Vein Tumor Thrombus			128 (44.4)	101 (49.0)	27 (32.9)	0.018*
Baseline AFP ≥ 400 ng/mL			120 (41.7)	89 (43.2)	31 (37.8)	0.43
BCLC:	A		4 (1.4)	3 (1.5)	1 (1.2)	0.007*
	B		74 (25.7)	42 (20.4)	32 (39.0)	
	C		210 (72.9)	161 (78.2)	49 (59.8)	
ECOG PS:	0		209 (72.6)	152 (73.8)	57 (69.5)	0.21
	1		70 (24.3)	50 (24.3)	20 (24.4)	
	2		9 (3.1)	4 (1.9)	5 (6.1)	
ICI:	Atezolizumab		47 (16.3)	36 (17.5)	11 (13.4)	0.48
	Nivolumab		223 (77.4)	155 (75.2)	68 (82.9)	0.86
	Pembrolizumab		18 (6.3)	15 (7.3)	3 (3.7)	0.30
ICI Monotherapy			173 (60.1)	119 (57.8)	54 (65.9)	0.23
ICI + Antiangiogenic Agent			113 (39.2)	85 (41.3)	28 (34.1)	0.29
Dual ICI (Nivolumab + Ipilimumab)			2 (0.7)	2 (1.0)	0 (0.0)	1
Prior Resection			58 (20.1)	45 (21.8)	13 (15.9)	0.33
Prior LRT (incl. SBRT)			171 (59.4)	128 (62.1)	43 (52.4)	0.14
Concurrent LRT (incl. SBRT)			126 (43.8)	92 (44.6)	34 (41.5)	0.69
Post-ICI therapy:			63 (21.9)	49 (23.8)	14 (17.1)	0.27
		Lenvatinib	30 (47.6)	25 (51.0)	5 (35.7)	
		Sorafenib	12 (19.0)	8 (16.3)	4 (28.6)	
		Ramucirumab	8 (12.7)	7 (14.3)	1 (7.1)	
		Bevacizumab only	9 (14.3)	6 (12.2)	3 (21.4)	
		Regorafenib	2 (3.2)	2 (4.1)	0 (0.0)	
		Cabozantinib	2 (3.2)	1 (2.0)	1 (7.1)	

^*^Statistically significant (*P* value < 0.05).

*HBV* hepatitis B virus, *HCV* hepatitis C virus, *EtOH* alcohol use, *NASH* non-alcoholic steatohepatitis, *AFP* alpha fetoprotein, *BCLC* Barcelona Clinic Liver Cancer, *ECOG* Eastern Cooperative Oncology Group, *PS* performance status, *ICI* immune checkpoint inhibitor, *LRT* locoregional therapy, *SBRT* stereotactic body radiation therapy.

Overall, patients with viral and non-viral etiologies of HCC were fairly well-balanced. However, compared to patients with non-viral HCC, patients with viral HCC were younger (median age 63.5 vs. 68 years, *P* < 0.001) and were more likely to have cirrhosis (91.3% vs. 75.6%, *P* < 0.001) but also CP class A liver disease (71.8% vs. 57.3%, *P* = 0.025). At the start of ICI therapy, patients with viral HCC also tended to have more advanced disease, with BCLC stage C (78.2% vs. 59.8%, *P* = 0.007) and higher rates of PVTT (49.0% vs. 32.9%, *P* = 0.018). No differences were observed in the rates of extrahepatic metastases, elevated AFP level, ECOG PS, or treatment received.

Given the strong influence of CP class B on treatment outcomes, patients with CP class A only liver disease were analyzed as a separate cohort, and these patient characteristics are reported in Supplementary Table 1. Of these 195 patients, 148 had viral HCC (75.9%) and 47 (24.1%) had non-viral HCC. The groups were well-balanced, and in this cohort, patients with viral HCC were also more likely to be younger (median age 63 vs. 68, *P* < 0.001) and to have cirrhosis (89.9% vs. 66.0%, *P* < 0.001). However, according to etiology, patients with CP class A disease did not differ in ECOG PS, type of therapy received, or disease burden, as measured by presence of PVTT, extrahepatic metastases, and baseline AFP levels.

### Survival outcomes by etiology

**Fig. 1:**
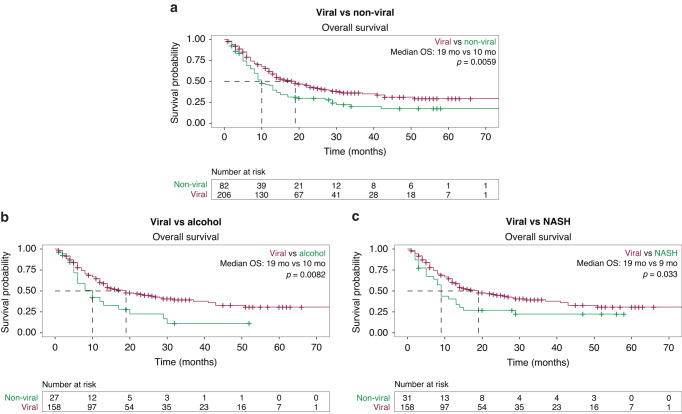
Overall survival based on etiology of HCC for the full cohort. **a** Viral vs. Non-Viral. **b** Viral vs. Alcohol. **c** Viral vs. Non-Alcoholic Steatohepatitis (NASH).

For the full cohort, after a median follow-up of 12.8 months (IQR: 6.4-25.0 months), the median OS (mOS) was 14 months (95% CI: 13–19 months). Patients with viral HCC had a mOS of 19 months (95% CI: 14–26 months) while patients with non-viral HCC had a mOS of 10 months (95% CI: 8-14 months; *P* = 0.006; Fig. 1a). Among patients with non-viral HCC, those with alcohol-related HCC (mOS 10 months; 95% CI: 6–20 months; *P* = 0.008; Fig. 1b) and NASH-associated HCC (mOS 9 months; 95% CI: 7–15 months; *P* = 0.033; Fig. 1c) both had worse OS than patients with viral HCC. While in univariate analysis non-viral etiology of HCC was associated with a worse OS (HR 1.6; 95% CI: 1.1–2.1; *P* = 0.006), this effect was not seen in multivariate analysis (HR 1.3; 95% CI: 0.96–1.8; *P* = 0.09; Table 2). ECOG PS ≥ 1 (HR 1.5; 95% CI: 1.1–2; *P* = 0.018) and CP class B (HR 2; 95% CI: 1.5–2.7; *P* < 0.001) were both correlated with worse OS in univariate analysis, but only CP class B was independently prognostic factor of worse OS in multivariate analysis (HR 1.7; 95% CI: 0.59–1.22; *P* = 0.002). Front-line ICI plus an AA was associated with better OS than ICI monotherapy in univariate (HR 0.57; 95% CI: 0.37–0.86; *P* = 0.008) but not multivariate analyses (HR 0.71; 95% CI: 0.46-1.1; *P* = 0.13; Table 2).

**Table 2:** Univariate and multivariate Cox regression analyses for the full cohort (*N* = 288).

	Univariate OS		Multivariate OS		Univariate PFS		Multivariate PFS	
	HR; 95% CI	*P* value	HR; 95% CI	*P* value	HR; 95% CI	*P* value	HR; 95% CI	*P* value
Age ≥ median vs. < median	0.83; 0.62–1.1	0.21			0.9; 0.7–1.2	0.40		
Gender Male vs. Female	0.79; 0.52–1.2	0.30			0.99; 0.7–1.4	0.97		
Cirrhosis Yes vs. No	1.1; 0.7–1.7	0.66			0.89; 0.61–1.3	0.53		
Tumor Size ≥ median vs. < median	1.2; 0.91–1.7	0.18			1.1; 0.84–1.4	0.54		
Extrahepatic Yes vs. No	0.92; 0.68–1.3	0.61			0.94; 0.73–1.2	0.67		
PVTT Yes vs. No	1.2; 0.91–1.7	0.17			0.98; 0.76–1.3	0.88		
PVTT Class vp 3–4 vs. vp 0–2	1.2; 0.85–1.6	0.89			1.0; 0.8–1.3	0.79		
MVI Yes vs. No	1.3; 1–1.8	0.051			1.0; 0.81–1.3	0.75		
ECOG PS ≥ 1 vs. 0	1.5; 1.1–2.0	0.018*	1.2; 0.86-1.7	0.28	1.5; 1.1–2	0.005*	1.2; 0.93–1.7	0.14
BMI ≥ 25 vs. <25	0.98; 0.72–1.3	0.90			1.0; 0.79–1.3	0.83		
BCLC Stage C vs. A/B	1.3; 0.88–1.8	0.20			0.99; 0.75–1.3	0.96		
AFP < 400 vs. ≥400	1.0; 0.75–1.4	0.90			0.96; 0.74–1.2	0.75		
Child Pugh Class B vs. A	2.0; 1.5–2.7	<0.001*	1.7; 0.59–1.2	0.002*	1.7; 1.3–2.2	<0.001*	1.4; 1.0–1.9	<0.001*
Etiology Non-Viral vs. Viral	1.6; 1.1–2.1	0.006*	1.3; 0.96–1.8	0.09	1.5; 1.2–2	0.002*	1.3; 0.97–1.7	0.078
ICI Regimen Combo vs. Mono	0.57; 0.37–0.86	0.008*	0.71; 0.46-1.1	0.13	0.58; 0.42–0.8	0.001*	0.69; 0.49–0.98	0.036*
Concurrent LRT Yes vs. No	1.3; 0.95–1.7	0.11			1.1; 0.87–1.4	0.38		

*Statistically significant (*P* value < 0.05).

*OS* overall survival, *PFS* progression-free survival, *HR* hazard ratio, *CI* confidence interval, *PVTT* portal vein tumor thrombosis, *MVI* macrovascular invasion, *ECOG PS* Eastern Cooperative Oncology Group performance status, *BMI* body mass index, *BCLC* Barcelona Clinic Liver Cancer, *AFP* alpha fetoprotein, *ICI* immune checkpoint inhibitor, *LRT* locoregional therapy.

**Fig. 2:**
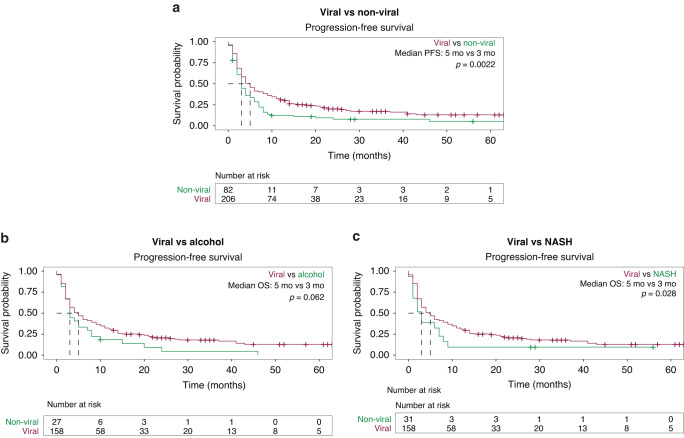
Progression-free survival based on etiology of HCC for the full cohort. **a** Viral vs. Non-Viral. **b** Viral vs. Alcohol. **c** Viral vs. Non-Alcoholic Steatohepatitis (NASH).

The median PFS (mPFS) for the cohort was 4 months (95% CI: 3–5 months). Patients with viral HCC had a longer mPFS than patients with non-viral HCC (5 vs. 3 months; *P* = 0.002; Fig. 2a). Patients with alcohol-related HCC had a non-significant worse mPFS than patients with viral HCC (3 vs. 5 months; *P* = 0.062; Fig. 2b). However, NASH-induced HCC correlated with a significantly worse mPFS (3 months; 95% CI: 2–7 months; *P* = 0.028; Fig. 2c). In univariate analysis, ECOG PS ≥ 1 (HR 1.5; 95% CI: 1.1–2; *P* = 0.005), CP class B (HR 1.7; 95% CI: 1.3–2.2; *P* < 0.001), and non-viral etiology of HCC (HR 1.5; 95% CI: 1.2–2; *P* = 0.002) were associated with worse PFS, but only CP class B remained independently prognostic of worse PFS in multivariate analysis (HR 1.4; 95% CI: 1.04–1.86; *P* < 0.001; Table 2). Combination ICI and an AA was correlated with better PFS in both univariate (HR 0.58; 95% CI: 0.42–0.8; *P* = 0.001) and multivariate (HR 0.69; 95% CI: 0.49–0.98; *P* = 0.036) analyses (Table 2). Post-ICI therapy was assessed (Table 1), and 63 patients (21.9%) received next line therapy. There was no statistical difference in the rate of post-ICI therapy based on etiology (23.8% vs. 17.1%, *P* = 0.27). The most common post-ICI agent for both groups was lenvatinib (47.6%), followed by sorafenib (19.0%). The etiology of HCC was not associated with the choice of next line AAs.

### Survival outcomes by etiology for Child Pugh class A liver disease

**Fig. 3:**
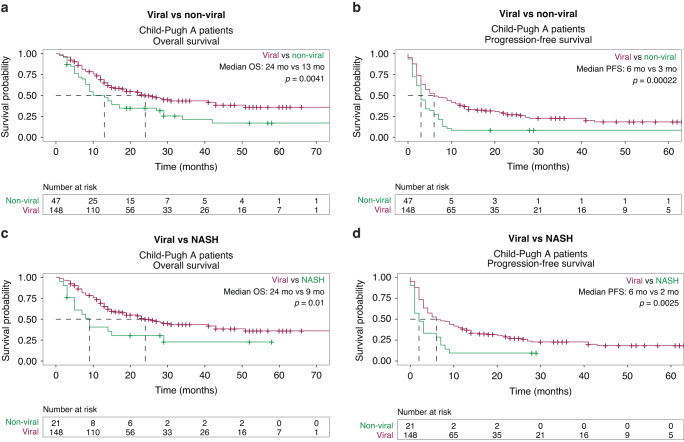
Survival based on etiology of HCC for Child Pugh class A patients only. **a** Overall Survival for Viral vs. Non-Viral. **b** Progression-Free Survival for Viral vs. Non-Viral. **c** Overall Survival for Viral vs. Non-Alcoholic Steatohepatitis (NASH). **d** Progression-Free Survival for Viral vs. Non-Alcoholic Steatohepatitis (NASH).

Given the strong negative prognostic value of CP class B and the fact that ICI trials only included CP class A patients [4, 6, 20], we performed separate analyses evaluating only patients with CP class A liver disease. After a median follow-up of 15.1 months (IQR: 8.6–28.4 months), the mOS (24 months; 95% CI 19–43 months) for patients with viral HCC, CP class A liver disease, and treated with front-line ICI was significantly better than the mOS of patients with non-viral HCC (13 months; 95% CI: 9–27 months; *P* = 0.003; Fig. 3a). Similarly, patients with viral HCC also had significantly longer mPFS (6 months vs. 3 months; *P* < 0.001; Fig. 3b). Non-viral HCC was associated with worse OS in univariate analysis (HR 1.8; 95% CI: 1.2–2.7; *P* = 0.005). No other prognostic factors for OS were identified in the CP class A cohort (Table 3). However, both ECOG PS ≥ 1 (HR 1.49; 95% CI: 1.02–2.2; *P* = 0.038) and non-viral HCC (HR 1.90; 95% CI: 1.32–2.7; *P* < 0.001) were independently associated with shorter PFS (Table 3). The combination of ICI and an AA prolonged PFS compared to ICI monotherapy only in univariate (HR 0.65; 95% CI: 0.46–0.94; *P* = 0.02) but not in multivariate analyses (HR 0.73; 95% CI: 0.51–1.1; *P* = 0.096). Among CP class A patients, the proportion of patients who received post-ICI therapy was balanced between viral vs. non-viral HCC groups (24.3% vs. 21.3%, *P* = 0.84; Supplementary Table 1). The choice of next line agent did not differ by etiology.

**Table 3:** Univariate and multivariate Cox regression analyses for the Child Pugh class A patients only (*N* = 195): Non-Viral (*N* = 47), Viral (*N* = 148).

	Univariate OS*		Univariate PFS		Multivariate PFS	
	HR; 95% CI	*P* value	HR; 95% CI	*P* value	HR; 95% CI	*P* value
Age ≥ median vs. < median	0.79; 0.54–1.1	0.21	0.91; 0.66–1.2	0.55		
Gender Male vs. Female	0.83; 0.49–1.4	0.50	1.0; 0.67–1.6	0.93		
Cirrhosis Yes vs. No	0.95; 0.57–1.6	0.84	0.81; 0.53–1.2	0.34		
Tumor Size ≥ median vs. < median	1.2; 0.79–1.7	0.45	1.0; 0.72–1.4	0.99		
Extrahepatic Yes vs. No	1.0; 0.69–1.5	0.96	1.0; 0.73–1.4	0.94		
PVTT Yes vs. No	1.3; 0.86–1.8	0.25	0.93; 0.67–1.3	0.65		
PVTT Class vp 3–4 vs. vp 0–2	1.1; 0.75–1.7	0.56	0.98; 0.70–1.4	0.91		
MVI Yes vs. No	1.3; 0.91–1.9	0.15	1.0; 0.73–1.4	0.98		
ECOG PS ≥ 1 vs. 0	1.2; 0.75–1.9	0.35	1.5; 1.0–2.2	0.034**	1.5; 1.0–2.2	0.038**
BMI ≥ 25 vs. < 25	0.96; 0.65–1.4	0.85	1.1; 0.82–1.6	0.45		
BCLC Stage C vs. A/B	1.2; 0.78–1.9	0.41	0.94; 0.66-1.4	0.75		
AFP < 400 vs. ≥ 400	1.2; 0.8–1.7	0.41	0.96; 0.69–1.3	0.79		
Etiology Non-Viral vs. Viral	1.8; 1.2–2.7	0.005**	2.0; 1.4–2.8	<0.001**	1.9; 1.3–2.7	<0.001**
ICI Regimen Combo vs. Mono	0.7; 0.47–1.2	0.18	0.65; 0.46–0.94	0.020**	0.73; 0.51–1.1	0.096
Concurrent LRT Yes vs. No	1.3; 0.86–1.8	0.24	1.1; 0.81–1.5	0.49		

*None of the variables was independently associated with overall survival in the multivariate analysis

**Statistically significant (*P* value < 0.05)

*OS* overall survival, *PFS* progression-free survival, *HR* hazard ratio, *CI* confidence interval, *PVTT* portal vein tumor thrombosis, *MVI* macrovascular invasion, *ECOG PS* Eastern Cooperative Oncology Group performance status, *BMI* body mass index, *BCLC* Barcelona Clinic Liver Cancer, *AFP* alpha fetoprotein, *ICI* immune checkpoint inhibitor, *LRT* locoregional therapy.

To better characterize the influence of NASH on survival outcomes in patients with CP class A liver disease, we compared the OS and PFS of patients with NASH to those with viral etiologies of HCC. NASH-related HCC was significantly associated with worse mOS compared to viral HCC (9 vs. 24 months; *P* = 0.01; Fig. 3c), which was confirmed in univariate analysis (HR 2.0; 95% CI: 1.2–3.; *P* = 0.012). No other variables were associated with OS (Supplementary Table 2). In addition, NASH-induced HCC had a significantly shorter mPFS than viral HCC (2 months vs. 6 months; *P* = 0.003; Fig. 3d) in the CP class A cohort. ECOG PS ≥ 1 (HR 1.52; 95% CI: 1.0–2.3, *P* = 0.04) and NASH-related HCC (HR 2.10; 95% CI: 1.3–3.4, *P* = 0.003) were independently prognostic of worse PFS in univariate and multivariate analyses (Supplementary Table 3). Combination therapy with an ICI and an AA was also independently associated with longer PFS (HR 0.67; 95% CI: 0.45–0.99; *P* = 0.04).

### Imaging responses based on etiology

Responses to ICI are described in Supplementary Table 4. In the full cohort of patients with evaluable responses by mRECIST criteria, the ORR was 29%. Patients with viral HCC had a higher ORR than patients with non-viral HCC that was not statistically significant (32% vs. 20%, *P* = 0.056). Patients with NASH in the full cohort had a similar ORR (22%, 8/37) as the non-viral group as a whole. There was no difference in DOR between patients who responded to ICI based on etiology, though only 16 patients with non-viral HCC responded. When only patients with CP class A liver disease were examined, patients with viral HCC had a significantly higher ORR than those with non-viral HCC (38% vs. 16%, *P* = 0.001). Patients with NASH-induced HCC and CP class A disease had an ORR of 15% (4/26), similar to the non-viral group. Again, no significant difference in DOR between the viral and non-viral HCC was observed, but only 6 patients with non-viral HCC could be included in the analysis.

Patients who received a combination of ICI and an AA experienced an ORR of 50%, which was significantly higher than the ORR of those who received ICI monotherapy (22%, *P* < 0.001), but there was no difference in DOR (Supplementary Table 5). Similar results were observed in patients with CP class A liver disease (Supplementary Table 5). Although no statistically significant difference was seen in ORR based on viral (27/48, 56%) vs. non-viral etiology (3/12, 25%, *P* = 0.10), only 3 patients with non-viral HCC received combination therapy, limiting the conclusiveness of these results.

### Outcomes and responses with receipt of concurrent locoregional therapy

We evaluated the outcomes of patients (*N* = 126, 43.8%) who received LRT within 30 days before or after initiation of ICI therapy. The baseline characteristics of patients who received ICI therapy alone and those who received concurrent LRT are described in Supplementary Table 6. The two cohorts were overall well-balanced, except that patients who had upfront systemic therapy alone were more likely to have extrahepatic metastases and prior resection, and less likely to have PVTT (Supplementary Table 6). Of 126 total patients, 92 (44.6%) had viral HCC and 34 (41.5%) had non-viral HCC (*P* = 0.69; Table 1). In the CP class A cohort, 88 patients (45.1%) had concurrent LRT, and similarly, rates of concurrent LRT did not differ based on etiology (Supplementary Table 1). Adding concurrent LRT to front-line ICI did not prolong mOS (16 vs. 13 months, *P* = 0.11), and there appeared to be a non-significant trend toward worse OS (Supplementary Fig. 2A). Similarly, concurrent LRT did not confer a PFS benefit to front-line ICI alone (mPFS 4 months for both; *P* = 0.33; Supplementary Fig. 2B). These findings are confirmed using Cox regression analyses in Table 2. In the CP class A only cohort, receipt of concurrent LRT also failed to affect OS (HR 1.3; 95% CI: 0.86-1.8; *P* = 0.24) and PFS (HR 1.1; 95% CI: 0.81-1.5; *P* = 0.49; Table 3). Notably, the presence and extent of PVTT did not influence the survival outcomes of treatment with concurrent LRT (Supplementary Fig. 3). In addition, in the full cohort, adding concurrent LRT did not improve ORR or DOR (Supplementary Table 7). In the CP class A cohort, treatment with an ICI alone was in fact associated with a longer DOR than treatment with concurrent LRT (42 vs. 8 months; *P* = 0.014).

## Discussion

While the standard front-line treatment for unresectable HCC is now immunotherapy, it has also become increasingly clear that not all patients benefit from this therapy. Because the etiology of HCC often confers unique characteristics to the tumor microenvironment [1], we aimed to assess whether a viral or non-viral cause of HCC could predict treatment outcomes with ICIs using a large cohort at a high-volume institution. We found that patients with viral HCC appeared to have better OS and PFS than patients with non-viral HCC, but etiology did not appear to be an independent prognostic factor for survival, except for patients with CP class A liver disease. Combination therapy with ICI plus AA may also be associated with improved OS and PFS, but it was inconsistently an independent prognosticator of survival. Interestingly, our study found that addition of concurrent LRT to first-line ICI did not significantly change the OS and PFS compared to treatment with ICI alone but may instead signal worse outcomes.

These findings agree with other studies assessing the impact of etiology on response to ICI in HCC, and our real-world experience reflects patterns observed in clinical trials. A meta-analysis of three randomized phase III trials of ICIs in advanced HCC using nivolumab [20], atezolizumab plus bevacizumab [4], and pembrolizumab [21], showed that first-line ICI improved survival in patients with HBV- or HCV-related HCC, but not in patients with non-viral HCC [10]. A recent retrospective propensity score matching analysis of patients with non-viral, unresectable HCC found that lenvatinib was associated with a longer OS and PFS than atezolizumab plus bevacizumab [22]. However, the observation that non-viral HCC portends a worse outcome with ICI has not been fully consistent across the existing literature. An updated meta-analysis from the same group with five randomized clinical trials of ICIs in advanced HCC [9] and a more recent abstract reported results from eight ICI trials [23] found conflicting results. A retrospective study of 323 Japanese patients with advanced HCC treated with atezolizumab plus bevacizumab found no difference in 12-month OS, PFS, ORR, or disease control rate based on etiology [24]. Our own study found that non-viral HCC was associated with worse survival outcomes and response to front-line ICI therapy, but these effects were most prominent for patients with CP class A liver disease. Although it is unclear why differences based on etiology were more consistently observed in patients with preserved liver function, CP class B liver disease itself being strongly and independently prognostic of worse outcomes suggests the presence of other confounders. Taken together, while etiology of HCC may influence ICI treatment outcomes, there are likely additional factors at play.

The type of non-viral etiology itself is important, and NASH specifically appears to promote an immune-suppressive tumor microenvironment that can confer ICI resistance [10]. Mechanisms proposed include: (1) exhausted and aberrantly activated CD8^+^PD1^+^ T cells in NASH livers cause tissue damage and disrupt tumor immune surveillance [10], (2) NASH-induced HCC have enriched Wnt and TGF-β signaling [25] implicated in ICI resistance [26, 27], and (3) patients responding to ICI have markers of pre-existing immunity and less regulatory T cells and oncofetal gene expression [28]. Interestingly, CD8^+^PD1^+^ T cells were not consistently observed in the non-viral population, indicating that further mechanistic studies are needed. Additionally, the effect of NASH on ICI efficacy is further obfuscated by the lack of specific non-viral etiology reporting in clinical trials. In fact, none of more than 30 clinical trials in advanced HCC captures NASH in their description of baseline patient characteristics, precluding the use of these phase III studies to address the impact of NASH. Even a recent post-hoc analysis assessing the response of atezolizumab plus bevacizumab in patients with NASH after manually re-assigning this etiology [29] has been challenged based on the initial allocation of etiology by the authors of IMbrave150 [30]. Our study is one of the first to attempt to fill this gap in knowledge. We found that patients with NASH-induced HCC likely drove the negative prognostic effects seen in the non-viral HCC group, particularly in patients with preserved liver function. Nevertheless, the small number of NASH patients limits our ability to draw firm conclusions. Future studies, particularly clinical trials, should stratify patients into specific etiologies to clarify how NASH affects treatment outcomes.

We also assessed whether an AA added benefit to immunotherapy. Inhibition of VEGF signaling has been hypothesized to reduce immunosuppression, normalize aberrant tumor vasculature, and improve T cell tumor infiltration [31, 32]. In our study, combination ICI plus AA was associated with better OS and PFS in univariate analyses, but it was only independently associated with better PFS but not OS in multivariate analyses. The combination had better ORR than ICI monotherapy, but there was no difference in DOR. In addition, we found no significant difference in ORR between patients with viral vs. non-viral HCC treated with the combination therapy, which is consistent with recent data, including IMbrave150 [5]. The mixed results are in line with findings from recent phase III clinical trials combining an ICI with an AA in advanced HCC, such as cabozantinib plus atezolizumab (improved PFS but not OS) [33], camrelizumab plus apatinib (longer PFS and OS) [34], and LEAP-002 with pembrolizumab plus lenvatinib (no OS or PFS benefit) [35]. Our study shows that AAs may confer additional benefit than ICIs alone, potentially regardless of HCC etiology, but larger prospective studies are needed to determine whether the combination can overcome ICI resistance often seen with non-viral HCC.

LRT is commonly used for limited BCLC stage B disease [2], but there is now increasing interest in combining LRT with front-line ICI. Previously, TACE plus lenvatinib improved OS, PFS, and ORR compared to lenvatinib alone [36]. In treating HCC with PVTT, TACE, TARE, RFA, and SBRT have all shown efficacy and safety [37]. However, the clinical trials investigating the combination of LRT with front-line ICI are ongoing [38]. There is preclinical evidence that ablative therapy can promote an anti-tumor immune response that an ICI could then augment [38, 39]. However, our study demonstrated no survival benefit with concurrent LRT, but rather, it trended toward worse outcomes. We acknowledge that this data is subject to selection bias as the decision to offer LRT was based on multidisciplinary tumor board consensus and could favor patients with liver-limited HCC, adequate liver function, and PVTT. To account for any bias, we demonstrated that rates of concurrent LRT receipt and outcomes were comparable between the full and CP class A only cohorts and that PVTT class did not have a significant effect on survival with or without concurrent LRT.

Other limitations include those inherent to retrospective cohort studies, including the presence of clinical heterogeneity when various ICIs and AAs were evaluated together. Our data also derived from a single institution with a preponderance of patients with viral-induced HCC and fewer with NASH-induced HCC. Although this distribution accurately reflects our patient population, expanding the cohort to include multi-institutional and international populations would strengthen our conclusions. While we observed an association of NASH-related HCC with worse outcomes compared to viral HCC, our sample size was small, and these results need to be confirmed prospectively with a larger cohort. There were also high rates of cirrhosis in the patient cohort, which could be reflective of both the clinical and radiologic definition of cirrhosis used and the fact that the study was conducted at a high-volume liver transplant center. We also included combination therapies including AAs and LRT, which are part of our routine clinical practice, but we attempted to reduce the potential for bias by analyzing outcomes separately based on treatment and by using multivariate analyses. However, dividing analyses according to each treatment group (ICI monotherapy, ICI plus AA, ICI plus LRT) substantially diminished the sample size of each group such that the contribution of etiology could not be meaningfully assessed. In addition, many patients underwent prior resection and/or LRT, which could have influenced survival outcomes. Future studies will be undertaken to specifically evaluate the efficacy of combination therapies in viral vs. NASH-induced and other non-viral HCC patients using a larger cohort.

To the best of our knowledge, our study is the first to compare the first-line ICI treatment outcomes of patients with advanced HCC based on etiology using a diverse population in the United States and separately analyze a more typical patient cohort with good liver function. We also assessed the impact of non-viral etiologies, particularly NASH, on clinical outcomes with more granularity. Additionally, we provided preliminary insight into the efficacy of various ICI combinations and modalities by leveraging our institutional expertise. Our study can be widely generalizable as it reflects outcomes in a real-world, high-volume cancer center that can be extrapolated to clinical practice at other institutions. Further, we collected detailed patient and tumor characteristics that allowed us to control for possible confounding factors. Finally, we used mRECIST to standardize response evaluations.

To conclude, in our cohort of patients with advanced HCC treated with ICI, non-viral etiology of underlying liver disease was associated with worse OS, PFS, and ORR compared to viral etiologies, especially for patients with CP class A liver disease. While our results add to the body of evidence that etiology of HCC plays a role in immunotherapy outcomes, more studies are needed to better understand the underlying mechanisms, and larger, prospective studies should be conducted to validate these outcomes, with particular attention to stratification of patients with NASH-induced HCC. Finally, we present preliminary results suggesting that the addition of an AA but not concurrent LRT may enhance ICI efficacy, which should also be further investigated in prospective studies.

## Supplementary information

Supplementary information

## Data Availability

Data supporting the findings of this study are available upon reasonable request.
