# Supplementary material for: Impact of underlying liver disease on unresectable hepatocellular carcinoma treated with immune checkpoint inhibitors
Source: BJC Rep. 2024 Jan 29;2:8. doi: 10.1038/s44276-024-00038-w (PMC11523972; doi:10.1038/s44276-024-00038-w)
Supplement: Supplementary file 1 — Supplementary information [file 44276_2024_38_MOESM1_ESM.docx]

**Supplemental Table 1.** Patient Demographics by Etiology, Child Pugh Class A Only (*N* = 195)

|  | | | **All Patients (%)** | **Viral (%)** | **Non-Viral (%)** | ***P* value** |
| --- | --- | --- | --- | --- | --- | --- |
| ***N*** | |  | 195 | 148 (75.9) | 47 (24.1) |  |
| **Age (years), median** | | | 64 | 63 | 68 | <0.001* |
| **Male** | | | 164 (84.5) | 123 (83.1) | 41 (87.2) | 0.65 |
| **Cirrhotic** | | | 164 (84.5) | 133 (89.9) | 31 (66.0) | <0.001* |
| **Diabetic** | | | 61 (31.3) | 37 (25.0) | 24 (51.1) | <0.001* |
| **Etiology:** | **HBV** | | 56 (28.7) | 38 (25.7) | N/A |  |
|  | **HCV** | | 151 (77.4) | 110 (74.3) | N/A |  |
|  | **EtOH** | | 41 (21.0) | 27 (18.2) | 14 (29.8) |  |
|  | **NASH** | | 32 (16.4) | 6 (4.1) | 26 (55.3) |  |
|  | **Other** | | 15 (7.7) | 14 (9.5) | 1 (2.1) |  |
|  | **None** | | 17 (8.7) | N/A | 11 (23.4) |  |
| **Measurable Viral Load** | | |  | 29 (19.6) | N/A |  |
| **Extrahepatic Metastasis** | | | 78 (40.0) | 60 (40.5) | 18 (38.3) | 0.86 |
| **Portal Vein Tumor Thrombus** | | | 78 (40.0) | 62 (41.9) | 16 (34.0) | 0.39 |
| **Baseline AFP ≥ 400 ng/mL** | | | 78 (40.0) | 62 (41.9) | 16 (34.0) | 0.39 |
| **BCLC:** | **A** | | 2 (1.0) | 2 (1.4) | 0 (0.0) | 0.088 |
|  | **B** | | 51 (26.2) | 33 (22.3) | 18 (38.3) |  |
|  | **C** | | 142 (72.8) | 113 (76.4) | 29 (61.7) |  |
| **ECOG PS:** | **0** | | 156 (80.0) | 115 (77.7) | 41 (87.2) | 0.40 |
|  | **1** | | 38 (19.5) | 32 (21.6) | 6 (12.8) |  |
|  | **2** | | 1 (0.5) | 1 (0.7) | 0 (0) |  |
| **Immunotherapy** | **Atezolizumab** | | 42 (21.5) | 33 (22.3) | 9 (19.1) | 0.84  0.20  0.12 |
|  | **Nivolumab** | | 138 (70.8) | 101 (68.2) | 37 (78.7) |  |
|  | **Pembrolizumab** | | 15 (7.7) | 14 (9.5) | 1 (2.1) |  |
| **ICI Monotherapy** | | | 105 (53.8) | 78 (52.7) | 27 (57.4) | 0.62 |
| **ICI + Antiangiogenic Agent** | | | 88 (45.1) | 68 (45.9) | 20 (42.6) | 0.74 |
| **Dual ICI (Nivolumab + Ipilimumab)** | | | 2 (1.0) | 2 (1.4) | 0 (0.0) | 1 |
| **Prior Resection** | |  | 50 (25.6) | 39 (26.4) | 11 (23.4) | 0.85 |
| **Prior LRT (incl. SBRT)** | | | 112 (57.4) | 89 (60.1) | 23 (48.9) | 0.18 |
| **Concurrent LRT (incl. SBRT)** | | | 88 (45.1) | 65 (43.9) | 23 (48.9) | 0.61 |
| **Post-ICI Therapy:** | | | 46 (23.6) | 36 (24.3) | 10 (21.3) | 0.84 |
|  | **Lenvatinib** | | 23 (50.0) | 19 (52.8) | 4 (40.0) |  |
|  | **Sorafenib** | | 11 (23.9) | 8 (22.2) | 3 (30.0) |  |
|  | **Ramucirumab** | | 6 (13.0) | 5 (13.9) | 1 (10.0) |  |
|  | **Bevacizumab only** | | 4 (8.7) | 3 (8.3) | 1 (10.0) |  |
|  | **Regorafenib** | | 1 (2.2) | 1 (2.8) | 0 (0.0) |  |
|  | **Cabozantinib** | | 1 (2.2) | 0 (0.0) | 1 (10.0) |  |

**Statistically significant (P value < 0.05)*

*HBV, hepatitis B virus; HCV, hepatitis C virus; EtOH, alcohol use; NASH, non-alcoholic steatohepatitis; AFP, alpha fetoprotein; BCLC, Barcelona Clinic Liver Cancer; ECOG, Eastern Cooperative Oncology Group; PS, performance status; ICI, immune checkpoint inhibitor; LRT, locoregional therapy; SBRT, stereotactic body radiation therapy*

**Supplemental Table 2.** Univariate Cox Regression Analyses for Overall Survival of the Child Pugh Class A Patients Only, Non-Alcoholic Steatohepatitis (NASH; *N* = 26) vs. Viral (*N* = 148)

|  | **Univariate*** | |
| --- | --- | --- |
|  | **HR; 95% CI** | ***P* value** |
| **Age**  ≥ median vs. < median | 0.83; 0.55-1.2 | 0.37 |
| **Gender**  Male vs. Female | 0.9; 0.51-1.6 | 0.71 |
| **Cirrhosis**  Yes vs. No | 1.0; 0.5-2.1 | 0.93 |
| **Tumor Size**  ≥ median vs. < median | 1.0; 0.67-1.6 | 0.89 |
| **Extrahepatic**  Yes vs. No | 1.0; 0.67-1.6 | 0.92 |
| **PVTT**  Yes vs. No | 1.2; 0.77-1.8 | 0.46 |
| **PVTT Class**  vp 3-4 vs. vp 0-2 | 1.1; 0.69-1.7 | 0.75 |
| **MVI**  Yes vs. No | 1.2; 0.79-1.8 | 0.40 |
| **ECOG PS**  ≥ 1 vs. 0 | 1.4; 0.84-2.2 | 0.20 |
| **BMI**  ≥ 25 vs. < 25 | 0.94; 0.61-1.4 | 0.77 |
| **BCLC Stage**  C vs. A/B | 1.2; 0.7-1.9 | 0.66 |
| **AFP**  <400 vs. ≥ 400 | 1.1; 0.72-1.7 | 0.66 |
| **Etiology**  NASH vs. Viral | 2.0; 1.2-3.6 | 0.012** |
| **ICI Regimen**  Combo vs. Mono | 0.69; 0.42-1.1 | 0.14 |
| **Concurrent LRT**  Yes vs. No | 1.1; 0.74-1.7 | 0.57 |

**None of the variables was independently associated with overall survival in the multivariate analysis*

***Statistically significant (P value < 0.05)*

*HR: hazard ratio; CI: confidence interval; PVTT: portal vein tumor thrombosis; MVI: macrovascular invasion; ECOG PS: Eastern Cooperative Oncology Group performance status; BMI: body mass index; BCLC: Barcelona Clinic Liver Cancer; AFP: alpha fetoprotein; ICI: immune checkpoint inhibitor; LRT: locoregional therapy*

**Supplemental Table 3.** Univariate and Multivariate Cox Regression Analyses for Progression-Free Survival of the Child Pugh Class A Patients Only, Non-Alcoholic Steatohepatitis (NASH; *N* = 26) vs. Viral (*N* = 148)

|  | **Univariate** | | **Multivariate** | |
| --- | --- | --- | --- | --- |
|  | **HR; 95% CI** | ***P* value** | **HR; 95% CI** | ***P* value** |
| **Age**  ≥ median vs. < median | 0.97; 0.69-1.4 | 0.86 |  |  |
| **Gender**  Male vs. Female | 1.1; 0.73-1.8 | 0.57 |  |  |
| **Cirrhosis**  Yes vs. No | 1.1; 0.61-2 | 0.75 |  |  |
| **Tumor Size**  ≥ median vs. < median | 0.86; 0.60-1.2 | 0.42 |  |  |
| **Extrahepatic**  Yes vs. No | 1.0; 0.70-1.4 | 0.99 |  |  |
| **PVTT**  Yes vs. No | 0.91; 0.65-1.3 | 0.61 |  |  |
| **PVTT Class**  vp 3-4 vs. vp 0-2 | 0.98; 0.69-1.4 | 0.93 |  |  |
| **MVI**  Yes vs. No | 0.96; 0.68-1.4 | 0.84 |  |  |
| **ECOG PS**  ≥ 1 vs. 0 | 1.6; 1.1-2.4 | 0.017* | 1.5; 1.0-2.3 | 0.04* |
| **BMI**  ≥ 25 vs. < 25 | 1.2; 0.83-1.7 | 0.33 |  |  |
| **BCLC Stage**  C vs. A/B | 0.91; 0.61-1.4 | 0.65 |  |  |
| **AFP**  <400 vs. ≥ 400 | 0.91; 0.64-1.3 | 0.59 |  |  |
| **Etiology**  NASH vs. Viral | 2.1; 1.3-3.5 | 0.003* | 2.1; 1.3-3.4 | 0.003* |
| **ICI Regimen**  Combo vs. Mono | 0.62; 0.42-0.92 | 0.016* | 0.67; 0.45-0.99 | 0.04* |
| **Concurrent LRT**  Yes vs. No | 1.1; 0.78-1.6 | 0.59 |  |  |

**Statistically significant (P value < 0.05)*

*HR: hazard ratio; CI: confidence interval; PVTT: portal vein tumor thrombosis; MVI: macrovascular invasion; ECOG PS: Eastern Cooperative Oncology Group performance status; BMI: body mass index; BCLC: Barcelona Clinic Liver Cancer; AFP: alpha fetoprotein; NASH: non-alcoholic steatohepatitis; ICI: immune checkpoint inhibitor; LRT: locoregional therapy*

**Supplemental Table 4.** Responses by Etiology

|  | **Viral** | **Non-Viral** | ***P* value** |
| --- | --- | --- | --- |
| **Full Cohort** | | | |
| **ORR** | 61/191 (32%) | 16/79 (20%) | 0.056 |
| **DOR, months** | 26 (95% CI: 17-NR) | 7 (95% CI: 3-NR) | 0.14 |
| **CP Class A Only** | | | |
| **ORR** | 53/139 (38%) | 6/46 (16%) | 0.001* |
| **DOR, months** | 35 (95% CI: 17-NR) | 6 (95% CI: 5-NR) | 0.88 |

**Statistically significant (P value < 0.05)*

*ORR: objective response rate; DOR: duration of response; CI: confidence interval; NR: not reached*

**Supplemental Table 5.** Responses by Receipt of Concurrent Antiangiogenic Therapy

|  | **ICI Only** | **ICI + AA** | ***P* value** |
| --- | --- | --- | --- |
| **Full Cohort** | | | |
| **ORR** | 47/210 (22%) | 30/60 (50%) | < 0.001* |
| **DOR, months** | 17 (95% CI: 7-NR) | 26 (95% CI: 17-NR) | 0.52 |
| **CP Class A Only** | | | |
| **ORR** | 30/56 (34%) | 30/56 (54%) | < 0.001* |
| **DOR, months** | 35 (95% CI: 17-NR) | 26 (95% CI: 17-NR) | 0.51 |

**Statistically significant (P value < 0.05)*

*ICI: immune checkpoint inhibitor; AA: antiangiogenic agent; ORR: objective response rate; DOR: duration of response; CI: confidence interval; NR: not reached*

**Supplemental Table 6.** Patient Demographics by Receipt of Locoregional Therapy (*N* = 288)

|  | | | **All Patients (%)** | **IO Only (%)** | **IO + LRT (%)** | ***P* value** |
| --- | --- | --- | --- | --- | --- | --- |
| ***N*** | |  | 288 | 162 (56.3) | 126 (43.8) |  |
| **Age (years), median** | | | 64 | 64 | 64 | 0.79 |
| **Male** | | | 245 (85.1) | 134 (82.7) | 111 (88.1) | 0.24 |
| **Cirrhotic** | | | 250 (86.8) | 136 (84.0) | 114 (90.5) | 0.12 |
| **Etiology:** | **HBV** | | 56 (19.4) | 29 (17.9) | 27 (21.4) | 0.69 (viral vs. non-viral) |
|  | **HCV** | | 151 (52.4) | 85 (52.5) | 67 (53.2) |  |
|  | **EtOH** | | 75 (26.0) | 38 (23.5) | 37 (29.4) |  |
|  | **NASH** | | 45 (15.6) | 23 (14.2) | 22 (17.5) |  |
|  | **Other** | | 16 (5.6) | 10 (6.2) | 6 (4.8) |  |
|  | **None** | | 17 (5.9) | 14 (8.6) | 3 (2.4) |  |
| **Child Pugh:** | **A** | | 195 (67.7) | 107 (66.0) | 88 (69.8) | 0.53 |
|  | **B** | | 93 (32.3) | 55 (34.0) | 38 (30.2) |  |
| **Extrahepatic Metastasis** | | | 105 (36.5) | 75 (46.3) | 30 (23.8) | <0.001* |
| **Portal Vein Tumor Thrombus** | | | 128 (44.4) | 61 (37.7) | 67 (53.2) | 0.017* |
| **Vp Class** | **0** | | 160 (55.6) | 101 (62.3) | 59 (46.8) | 0.26 (vp 0-2 vs. vp 3-4) |
|  | **1** | | 3 (1.0) | 2 (1.2) | 1 (0.8) |  |
|  | **2** | | 23 (8.0) | 9 (5.6) | 14 (11.1) |  |
|  | **3** | | 41 (17.7) | 25 (15.4) | 26 (20.6) |  |
|  | **4** | | 51 (17.7) | 25 (15.4) | 26 (20.6) |  |
| **Baseline AFP ≥ 400 ng/mL** | | | 120 (41.7) | 62 (38.3) | 58 (46.0) | 0.19 |
| **BCLC:** | **A** | | 4 (1.4) | 1 (0.6) | 3 (2.4) | 1 (A+B vs. C) |
|  | **B** | | 71 (24.7) | 41 (25.3) | 30 (23.8) |  |
|  | **C** | | 213 (74.0) | (74.1) | 93 (73.8) |  |
| **ECOG PS:** | **0** | | 209 (72.6) | 113 (69.8) | 96 (76.2) | 0.09 |
|  | **1** | | 70 (24.3) | 41 (25.3) | 29 (23.0) |  |
|  | **2** | | 9 (3.1) | 8 (4.9) | 1 (0.8) |  |
| **Immunotherapy** | **Atezolizumab** | | 47 (16.3) | 20 (12.3) | 27 (21.4) | 0.7625 |
|  | **Nivolumab** | | 223 (77.4) | 127 (78.4) | 96 (76.2) |  |
|  | **Pembrolizumab** | | 18 (6.3) | 15 (9.3) | 3 (2.4) |  |
| **ICI Monotherapy** | | | 223 (77.4) | 128 (79.0) | 95 (75.4) | 0.47 |
| **ICI + Antiangiogenic Agent** | | | 63 (21.9) | 33 (20.4) | 30 (23.8) | 0.48 |
| **Dual ICI (Nivolumab + Ipilimumab)** | | | 2 (0.7) | 1 (0.6) | 1 (0.8) | 0.86 |
| **Prior Resection** | |  | 58 (20.1) | 42 (25.9) | 16 (12.7) | 0.007* |
| **Prior LRT (incl. SBRT)** | | | 130 (45.1) | 80 (49.4) | 50 (39.7) | 0.10 |

**Statistically significant (P value < 0.05)*

*IO, immunotherapy; LRT, locoregional therapy; SBRT, stereotactic body radiation therapy*; *HBV, hepatitis B virus; HCV, hepatitis C virus; EtOH, alcohol use; NASH, non-alcoholic steatohepatitis; AFP, alpha fetoprotein; BCLC, Barcelona Clinic Liver Cancer; ECOG, Eastern Cooperative Oncology Group; PS, performance status; ICI, immune checkpoint inhibitor; SBRT, stereotactic body radiation therapy*

**Supplemental Table 7.** Responses by Receipt of Concurrent Locoregional Therapy

|  | **ICI without LRT** | **ICI + LRT** | ***P* value** |
| --- | --- | --- | --- |
| **Full Cohort** | | | |
| **ORR** | 48/148 (33%) | 28/122 (23%) | 0.080 |
| **DOR, months** | 33 (95% CI: 17-NR) | 8 (95% CI: 3-NR) | 0.052 |
| **CP Class A Only** | | | |
| **ORR** | 34/99 (34%) | 25/86 (29%) | 0.52 |
| **DOR, months** | 42 (95% CI: 26-NR) | 8 (95% CI: 5-NR) | 0.014* |

**Statistically significant (P value < 0.05)*

*ICI: immune checkpoint inhibitor; LRT: locoregional therapy; ORR: objective response rate; DOR: duration of response; CI: confidence interval; NR: not reached*

**Supplemental Figures**


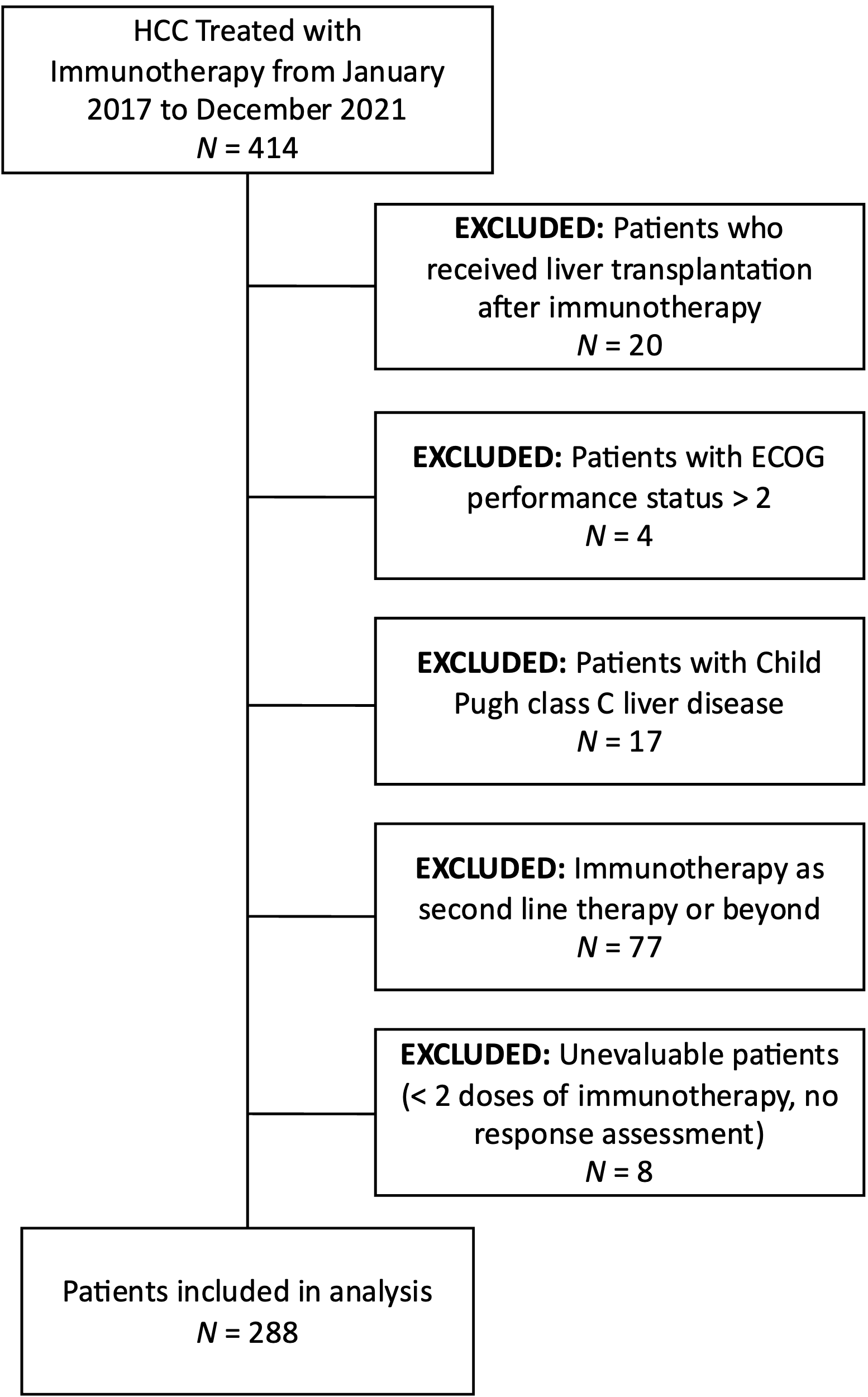


**Supplemental Figure 1:** CONSORT Diagram.

**Supplemental Figure 2:** Survival Based on Receipt of Concurrent Locoregional Therapy. (A) Overall Survival. (B) Progression-Free Survival

**Supplemental Figure 3:** Survival Outcomes with Concurrent Locoregional Therapy or Immunotherapy Alone Based on Portal Vein Tumor Thrombosis Class for the Full Cohort. (A) Overall Survival. (B) Progression-Free Survival
